# Proteomic Analysis of Human Brain Microvascular Endothelial Cells Reveals Differential Protein Expression in Response to Enterovirus 71 Infection

**DOI:** 10.1155/2015/864169

**Published:** 2015-03-02

**Authors:** Wenying Luo, Jiayu Zhong, Wei Zhao, Jianjun Liu, Renli Zhang, Liang Peng, Wenxu Hong, Sheng He Huang, Hong Cao

**Affiliations:** ^1^Department of Microbiology, School of Public Health and Tropical Medicine, Southern Medical University, Guangzhou 510515, China; ^2^Department of Clinical Laboratory, Affiliated Hospital of Guangdong Medical College, Zhanjiang 524001, China; ^3^Central Laboratory, Guangzhou Women and Children's Medical Center, Guangzhou 510623, China; ^4^Shenzhen Center for Disease Control and Prevention, Shenzhen 518055, China; ^5^Clinical Laboratory, The Second Affiliated Hospital of Guangzhou Medical College, Guangzhou 510260, China; ^6^Saban Research Institute, Children's Hospital Los Angeles, University of Southern California, Los Angeles, CA 90027, USA

## Abstract

2D DIGE technology was employed on proteins prepared from human brain microvascular endothelial cells (HBMEC), to study the differentially expressed proteins in cells at 0 h, 1 h, 16 h, and 24 h after infection. Proteins found to be differentially expressed were identified with matrix-assisted laser desorption/ionization time-of-flight/time-of-flight mass spectrometry (MALDITOF/TOF MS) analysis. We identified 43 spots showing changes of at least 2.5 fold up- or downregulated expressions in EV71-infected cells at different time when comparing to control, and 28 proteins could be successfully identified by MALDI TOF/TOF mass spectrometry analysis. 4 proteins were significantly upregulated, and 6 proteins were downregulated, another 18 proteins were different expression at different incubation time. We identified changes in the expression of 12 cellular metabolism-related proteins, 5 molecules involved in cytoskeleton, 3 molecules involved in energy metabolism, 2 molecules involved in signal transduction, 1 molecule involved in the ubiquitin-proteasome pathway, 1 molecule involved in cell cycle, 1 molecule involved in apoptosis-related protein, 1 molecular chaperone, and 2 unknown proteins. These findings build up a comprehensive profile of the HBMEC proteome and provide a useful basis for further analysis of the pathogenic mechanism that underlies EV71 infections to induce severe neural complications.

## 1. Introduction

Human enterovirus 71 (EV71) was first described during an outbreak with central nervous system complications in 1974 [1], which is a small, nonenveloped positive- stranded RNA virus that belonging to the enterovirus genus of the Picornaviridae family [2]. EV71 is a major pathogen of hand-foot-and-mouth disease (HFMD); however, there were greater numbers of fatal cases of HFMD with symptoms of central nervous system (CNS) occurred in Taiwan [3], western Australia [4], Malaysia [5], Japan [6], Singapore [7], and South Korea [8] during the last decade. Since 2008, in the mainland of China, large outbreaks of HFMD have been reported and resulted in millions of infections and hundreds of deaths in children. In China, it is reported that many cases associated aseptic meningitis complications infected by Enterovirus, most of which is EV71 pathogen [9].

Many data show that EV71 infection has a complex pathogenesis and that the central nervous system (CNS) is likely the main target of the EV71 virus [10]. The well-established murine and cynomolgus monkey models have demonstrated that EV71 infection may cause neurological lesions in the CNS [11, 12]; recently, EV71 Virus infected rhesus monkeys through four route can result in lesion in the CNS [13]. However, the mechanism that underlies EV71 infections to induce severe neural complications in patients still remains unknown. Therefore, it is very important to elucidate pathogenic mechanism of EV71 CNS infection.

The BBB which is primarily constituted by the brain capillary endothelial cells interacts with neighboring cells, such as pericytes, astrocytes, and neurons to maintain the barrier integrity [14–16]. The human brain microvascular endothelial cells (HBMEC) are a special type of cell that constitute the BBB [17]. Like poliovirus, EV71 transmitted by the fecal-oral route has an affinity for cells in the central nervous system (CNS) and manifests as poliomyelitis-like paralysis [18]. Proteomics analysis is currently considered to be a powerful tool for global evaluation of protein expression. In this study, proteomics analyses of HBMEC after EV71 infection were performed. First, we found EV71 could infect and replicate in HBMEC through detecting EV71 RNA [19]. Secondly, the changes progress of HBMEC proteome induced by EV71 was explored. We found EV71-infected HBMEC can induce 28 differently expressed protein spots compared with control. These proteins might affect BBB dysfunction and lead to the development of EV71 CNS disease.

## 2. Material and Methods

### 2.1. Materials

HBMEC was a generous gift from professor Huang, (University of Southern California, USA). Human embryonic rhabdomyosarcoma (RD) cells were kindly provided by the Centre for Disease Control and Prevention of Guangdong Province. The newly identified strain of EV71 (KC122766) was isolated from a rectal swab specimen of a 6-year-old male which was diagnosed as critical care HFMD with encephalitis complications. EV71 were amplified by using RD cells. Vero cells were conserved in our laboratory. DJ-1, vimentin, and heat shock proteins 27 rabbit anti-human polyclonal antibody were obtained from Santa Cruz Biotechnology (Santa, USA). All other chemicals in this study were analytical grade.

### 2.2. Preparation of Virus Stocks and Virus Titration

EV71 were amplified by using RD cells. Virus was propagated in RD cells which were maintained in DMEM supplemented with 10% heat inactivated FBS and antibiotics. Briefly, 80~90% confluent monolayers of RD cells were inoculated with the virus; once 90% of the cells showed cytopathic effect (CPE), the culture medium and cell debris were harvested and were repeated freezing and thawing three times, cell debris was removed by centrifugation at 1,000 g for 10 min. Supernatants were filtered through a 0.22 *μ*L membrane filter (Millipore, Bedford, MA) and stored at −80°C before use. Furthermore, the virus titers were determined by a plaque assay using vero cells as described in [20]. Plaques developed by series dilute suspension of EV71 virus in Vero cell cultures 96 h postinfection after crystal violet staining. Scoring was done by counting the plaques in each well.

### 2.3. Comparative Proteome Analyses of HBMEC Protein Expression in Response to EV71 Infection

#### 2.3.1. HBMEC Infection and Protein Extraction

HBMEC were first seeded in four 75 cm^2^ culture flasks. Then, cells were infected with EV71 Strain (KC122766) at a MOI of 5. As a negative control, one culture flask was mock infected with DMEM. Briefly, time was set as 0 h when HBMEC was mock infected with DMEM. Time was set as 1 h after HBMEC was infected with EV71 for 1 h. At 1 h, 16 h, and 24 h after infection, infected cells and mock infected cells (0 h) were harvested by scraping and washed with the PBS twice (0.01 M, pH 7.4) at 4°C. Cells were then pelleted by centrifuging at 4°C (150 g for 10 min), and 800 *μ*L ice-cold lysis buffer (containing 20 mM Tris base, 2 M Thiourea, 7 M Urea, and 2% Chaps) was added into the centrifuge tube for 60 min on ice. The protein extract supernatant was collected by centrifuging at 16,000 g for 1 h at 4°C. The proteins were separated into several equal parts (125 *μ*L) and were further purified by precipitation with chloroform/methanol as described [21] and stored at −80°C until further analysis.

#### 2.3.2. Two-Dimensional Gel Electrophoresis

The concentrations of the extracted proteins were measured with the Infinite M1000 spectrophotometer (Tecan, Switzerland) at 480 nm. Samples were then diluted to 1200 *μ*g with rehydration buffer (containing 2 M Thiourea, 7 M urea, 4% Chaps) and applied to 24 cm (pH 4–7) ReadyStrip IPG Strips. The strips were first rehydrated and then focused on the Ettan IPGphor 3 IEF system (GE, USA) with the following conditions: rehydrated for 12 h at 20°C (30 V), 300 V for 30 min with rapid ramping, 700 V for 30 min with rapid ramping, 1,500 V for 1.5 h, 9,000 V for 3 h with linear ramp, and finally 9,000 V for 4 h with rapid ramping. After equilibration with equilibration buffers (containing 100 mM DTT and 250 mM IAA), the gel strips were applied to second-dimensional SDS-PAGE for 45 min at 1 W/gel, then 4.5 h at 11 W/gel. The gels were fixed for at least 2 h with stationary liquid (containing 40% ethanol, 10% glacial acetic acid, and 68% (m/v, g/L) acetic acid sodium without water) and then stained with coomassie brilliant blue G-250 (1000 mL solution containing G-2501.2 g, ammonium sulfate 100 g, phosphoric acid 100 mL, methanol 200 mL) for 6 h and subsequently destained with distilled water until background staining were negligible. In addition, the fixed gels are also stained with MS compatible silver nitrate method [22]. The silver stained gels were used for analysis and coomassie blue stained gels were used for protein identification.

#### 2.3.3. Image Acquisition and Analysis

The gels were captured at 300 dpi with an Image Scanner (Amershan pharmacia biotech, Piscataway, NJ). The spot intensities were determined by Image Master 2D platinum 7.0 (GE). All the experiments were done in triplicates to ensure reproducibility. Statistical analysis of protein variations was carried out in 2D gels prepared from three replicates in each group. In each group, matched spots with coefficient variation less than 50% on vol. % were included for further between group analyses. Student's *t*-test analysis on vol. % of matched spots between groups was done to find out significant expressional changes (*P* < 0.05). Proteins determined to be differentially expressed were selected for MS identification.

#### 2.3.4. In-Gel Digestion and MAlDI-TOF–TOF-MS/MS

The protein spots that were significant (|ratio| > 2.5, *P* < 0.05) increased or decreased in samples compared with control samples were chosen for further analysis. The differential protein spots were excised from the gel and washed twice with water. The gels were then destained and washed in 25 mM NH_4_HCO_3_ and 50% (v/v) acetonitrile. Destaining was repeated two more times. The gel was subsequently dehydrated with 50 *μ*L acetonitrile and dried with a vacuum centrifuge. Digestion was done with 12.5 ng/*μ*L of sequencing grade modified trypsin in 25 mM ammonium bicarbonate and incubated for 30 min at 4°C, then 10 *μ*L 25 mM ammonium bicarbonate was added into each tube at 37°C for 18 h. The digested peptides were concentrated by centrifugation and were moved into another tube. The digested peptides were mixed with freshly prepared matrix solution (1 mg of HCCA in 0.3 mL of 0.1% TFA and 0.7 mL acetonitrile) in a 1 : 1 (v/v) ratio and applied onto a target plate (MTP 600-384 anchor chip). All samples were analyzed using an Autoflex MALDI-TOF MS (Bruker Daltonik, Bremen, Germany). Peptide tolerance was set at 100 ppm with fixed modification of cysteine carbamidomethyl, variable modification of methionine oxidated, and permitted missed cleavage of up to 1. Trypsin cleavage of the protein is at the C-terminal side of KR unless next residue is P. Protein identification was achieved by a protein blast search using the NCBI protein database (http://blast.ncbi.nlm.nih.gov/Blast.cgi). The significance of the change in spot intensities was analyzed by *χ*2 test with 2 degrees of freedom (*α* = 0.05).

#### 2.3.5. Validation of the MAlDI-TOF-TOF-MS/MS Results by Western Blot

Western blot analysis was performed to ensure the reliability of the MAlDI-TOF–TOF-MS/MS results. Selected differentially expressed proteins in cell samples were verified as previously reported [23]. HBMEC infection, protein extraction and protein concentrations were performed as previously described in this study. Rabbit anti-DJ-1, vimentin and heat shock proteins 27 polyclonal antibodies were primary antibodies for the immunodetection. HRP-conjugated goat-anti rabbit IgG was used as secondary antibody (Santa, USA). Tubulin and GAPDH (Santa, USA) was used as a loading control. Bands were visualized by chemiluminescence with ChemiDocXRC+ Imaging System (Bio-Rad, USA) using ECL detection reagents (GE Healthcare, USA). Quantification of the detected bands density value was performed using the version 4.6.2 Quantity One software (Bio-Rad, USA). All immunoblots were run at least in triplicate.

### 2.4. Statistical Analysis

All data in this study were presented as mean ± SD. All these statistical analyses were carried out using origin 7.5 or SPSS 13.0 for windows. Differences with *P* < 0.05 were considered to be statistically significant.

## 3. Results and Discussion

**Figure 1:**
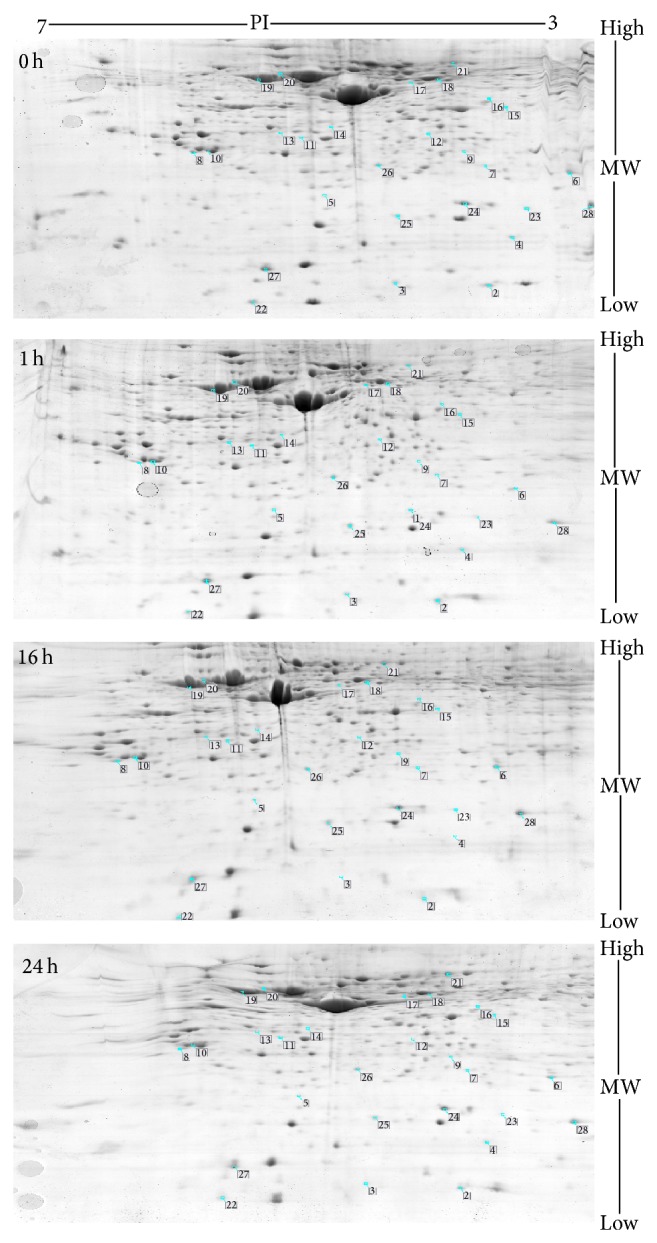
Analysis of HBMEC infected with EV71 as revealed by 2D DIGE analysis.

The proteins of HBMEC infected by EV71 extracts were prepared and explored the 2-DE analysis. Analysis of 2D images from protein lysates of EV71-infected and mock- infected HBMEC identified 1435 matching protein spots. There were 43 spots showing changes of at least 2.5 fold up- or downregulated expressions in EV71-infected samples at different time when comparing to control (Figure 1). Among the proteins with significant expression changes, 28 proteins could be successfully identified by MALDI TOF/TOF mass spectrometry analysis (Table 1) and 15 proteins could not be successfully identified. The reason that 15/43 spots were not identified might be that (1) the proteins might have characteristics of low abundance and (2) the differential protein spots might contain a variety of proteins. Of these 28 differentially expressed protein spots, 4 proteins were significantly upregulated, and 6 proteins were downregulated, and another 18 proteins were different expression at different incubation time. The four upregulated proteins were identified as protein expression at 1 hpi, 16 hpi, and 24 hpi upregulated after EV71 infection, which included splicing factor arginine/serine-rich 2, ubiquinol-cytochrome c reductase core I protein, glutathione synthetase, and actin cytoplasmic 1. The six downregulated proteins were identified as protein expression at 1 hpi, 16 hpi, and 24 hpi downregulated after EV71 infection, which included L-lactate dehydrogenase B chain OS, protein DJ-1, serine/threonine-protein phosphatase 6 catalytic subunit isoform b, spermidine synthase, Vimentin, and thymosin beta-10.

**Figure 2:**
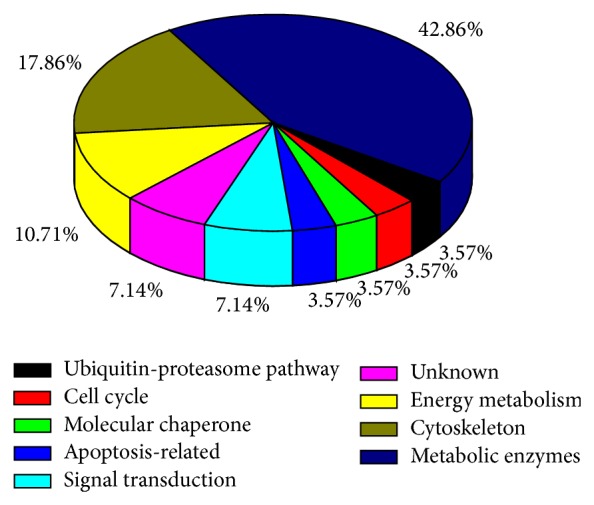
Classification analysis of 28 differentially expressed proteins found in EV71-infected HBMEC. Categorization was based on information that was obtained from the online PANTHER classification system.

According to cellular functions and processes, we identified changes in the expression of 12 cellular metabolism-related proteins (metabolic enzymes), 5 molecules involved in cytoskeleton, 3 molecules involved in energy metabolism, 2 molecules involved in signal transduction, 1 molecule involved in the ubiquitin-proteasome pathway, 1 molecule involved in cell cycle, 1 molecule involved in apoptosis-related proterin, 1 molecular chaperone, and 2 unknown proteins (Figure 2). Our studies showed that the expression of several enzyme molecules including deoxyuridine 5′-triphosphate nucleotidohydrolase, acyl- CoA-binding protein isoform 1, L-lactate dehydrogenase B chain OS, ubiquitin-conjugating enzyme E_2_N, uroporphyrinogen decarboxylase, ubiquinol-cytochrome c reductase core I protein, glutathione synthetase, and Inosine triphosphate pyrophosphatase were differentially regulated by EV71.

**Figure 3:**
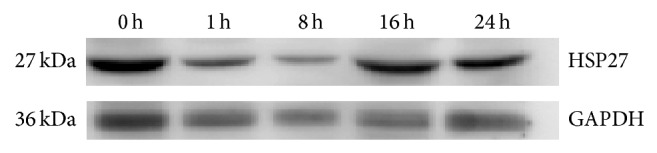
The expression of HSP27 in EV71-infected HBMEC by western blotting (0 h, Control; 1 h, 8 h, 16 h, and 24 h represented the time of EV71 infected HBMEC).

Heat shock proteins are the products of several distinct gene families that are required for cell survival during stress and are named according to the approximate relative molecular mass of their encoded proteins such as HSP27; heat shock proteins belong to molecular chaperones and which are reported to be crucial for virus propagation. HSP27 (spots 2) were observed to be downregulated at 1 hpi and 16 hpi, but upregulated at 24 hpi in the host cells infected with EV71. In order to correlate the protein expression level, we examine HSP27 protein by western blot analysis (Figure 3), from picture we can find that protein expression is downregulated at 1 hpi and 8 hpi compare with control, but is upregulated at 16 hpi and 24 hpi compare 1 hpi.

**Figure 4:**
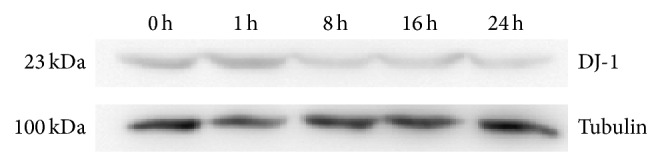
The expression of DJ-1 in EV71-infected HBMEC by western blotting (0 h, Control; 1 h, 8 h, 16 h, and 24 h represented the time of EV71 infected HBMEC).

Protein DJ-1, also called PARK7 protein, is encoded by the dj-1 gene, which was described to be involved in the antioxidant response of the cells and is a redox-responsive cytoprotective protein with diverse functions. DJ-1 protein was observed to be downregulated at 1 to 24 hpi. This may be due to redox potential of cells infected with EV71 is decreased; thus, the protein expression is decreased compared with control. We analyzed the DJ-1 protein by western blot analysis and found the protein is downregulated from 1 to 24 hpr (Figure 4).

Stathmin-1 was observed to be upregulated at 1 hpi and 16 hpi, but downregulated at 24 hpi in the host cells infected with EV71, Stathmin-1 is involved in the regulation of the microtubule filament system by destabilizing and preventing assembly of microtubules, Thus, its increased expression may be associated with destroying the cell in the early stage of EV71 infection. But the downregulation of Stathmin-1 at 24 hpi supported the previous findings by Leong and Chow when RD cells were infected with EV71 MS/7423/87 strain, which is expected to induce growth arrest in EV71-infected cells [24]. Deoxyuridine triphosphate nucleotidohydrolase (dUTPase) was responsible for maintaining low intracellular levels of dUTP, thus preventing the incorporation of dUTP into DNA [25, 26]. It widely exists in eukaryotic and prokaryotic cells, viruses, and other biological organisms [27]. Study demonstrated expression or the activity of dUTPase is related to EBV replication [28]. In this study, dUTPase only appeared at 1 hpi but was not detected at 0 h, 16 hpi, and 24 hpi. This showed that dUTPase might be related to the process of EV71 attaching and penetrating into HBMEC. But, its mechanism remains to be further studied. Acyl-CoA-binding protein (ACBP), a low molecular mass (m) (~10 kDa) soluble protein ubiquitous in eukaryotes, plays an important housekeeping role in lipid metabolism by maintaining the intracellular acyl-CoA pool. ACBP is involved in lipid biosynthesis and transport, gene expression, and membrane biogenesis [29]. In this study, ACBP was upregulated at 1 hpi and downregulated at 16 hpi and 24 hpi. Maybe the ACBP interact with EV71 and thus may facilitate EV71 entry.

**Figure 5:**
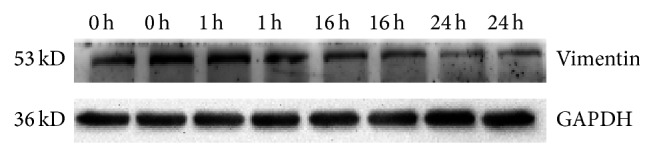
The expression of Vimentin in EV71-infected HBMEC by western blotting (0 h, Control; 1 h, 16 h, and 24 h respresented the time of EV71 infected HBMEC).

Our current study also demonstrated that several cytoskeleton proteins including vimentin, actin, cofilin-1, and thymosin beta-10 were differentially regulated. Actin was observed to be upregulated at 1 to 24 hpi. Studies demonstrate some viruses interact directly with the cytoskeletal transport machinery for intracellular transport. Study demonstrates cofilin-1 plays a central role in maintaining actin cytoskeletal dynamics by severing F-actin and allowing for reorganization and formation of new filaments [30–32]. The protein was also reported to be related to apoptotic cell death, cancer invasion, metastasis, and chemoresistance [33–36]. In this study, cofilin-1 was found to be downregulated at 1 hpi and upregulated at 8 hpi and 24 hpi. The upregulation of cofilin-1 in HBMEC at 24 hpi is accord with the previous findings on RD cells infected with EV71 MS/7423/87 strain [24]. We also demonstrated EV71 could lead to the apoptotic of HBMEC [37]. Therefore, we speculated the different expression of cofilin-1 in EV71-infected HBMEC might be involved in apoptotic cell death caused by EV71 infection: further studies focusing on the functional properties of cofilin-1 and predictive value for EV71 infection may permit identification of biomarkers for EV71 infection and development of new therapeutic methods. Vimentin is the major IF protein in mesenchymal cells, which mainly assigned functions in maintaining the structural and mechanical integrity of cells and also participates in a number of critical cellular processes such as adhesion, migration and cell signalling. Vimentin was observed to be downregulated at 1 to 24 hpi. This may be due to decreased expression of vimentin is associated with inhibiting the proliferation of HBMEC. Vimentin is mainly located in the cytoplasm, but cell surface-expressed vimentin has been reported as EV71 receptor in human astrocyte cell [38]. It was also found that vimentin expression was downregulated by western analysis at 1 hpi, 16 hpi, and 24 hpi. Vimentin and other cytoskeletal filaments have been shown to play important roles in virus entry and/or infection for many viruses, such as human immunodeficiency virus type 1, Japanese encephalitis virus, vaccinia virus, adenovirus type 2, herpes simplex virus type 1, hepatitis C virus [39–41]. The studies present here are the basis to understand the interaction between viruses and the cell cytoskeleton; however, further specific experiments will define the exact mechanism of cytoskeleton in EV71-infected HBMEC (Figure 5).

EV71 infection cause central nervous system (CNS) complications whether through BBB is unclear. Furthermore, EV71 invasion will modify the patterns of protein expression in HBMEC, which may affect the normal physiological function of HBMEC and determine its pathogenic progress and consequence. Therefore, studies on viral infections proteomics contributes to uncover the mechanism of interaction between EV71 and HBMEC and viral molecular pathogenesis, find early biomarker of EV71 infection, develop earlier diagnostic method, evaluate therapeutic effect and prognosis and so on. In this study, we identified 28 differentially expressed protein in HBMEC after EV71 infection, and western analysis three proteins. Those differential protein expression in EV71-infected HBMEC in vitro may not completely be according to the protein level in vivo. We regretted that most of the confirmed differential protein didnot be futher tested the expression level by western blotting, but our identified three proteins have be confirmed expression level by western blotting, which have important roles in virus infection; for example, HSP27 and vimentin are associated with virus entry and/or infection, and DJ-1 protein may associated with EV71 infection caused encephalitis. Our study on HBMEC proteome of BBB in CNS may contribute to understand the severity of EV71 brain encephalitis in clinic, and some differential proteins may provide a new way to find early biomarker candidate during EV71 infection developed severe case; our work on differential protein expression of HBMEC may provide a possible insight into central nervous system complications caused by EV71 virus infection, and find new drug candidate against EV71. This study builds up a comprehensive profile of the HBMEC proteome and provides a useful basis for further analysis of the pathogenic mechanism that underlies EV71 infections to induce severe neural complications.

## Figures and Tables

**Table 1:** Differential proteins identified by mass spectrometry (MS) after 2D DIGE of in HBMEC from EV71-infected contrast to mock-infected.

Spot number	Protein Name	Gene name	Accession number	Nominal mass (Mr)/calculated PI	Infection time (hpi)			Sequence coverage (%)
					1	16	24	
1	Deoxyuridine 5′-triphosphate nucleotidohydrolase	DUT	gi|3041664	26975/9.65	—	0	0	11
2		ACBP1	gi|10140853	11786/5.41	↑	↓	↓	34
3	L-lactate dehydrogenase B chain OS	LDHB	gi|291575128	36900/5.71	↓	↓	↓	8
4	Ubiquitin-conjugating enzyme E2 N	UBE2N	gi|4507793	17184/6.13	↑	↓	↑	19
5	Unnamed protein product	Unnamed gene product	gi|194375299	40819/5.78	↑	↑	↑	23
6	Protein DJ-1	DJ-1, PARK7	gi|31543380	20050/6.33	↓	↓	↓	20
7	Microtubule-associated protein RP/EB family member 1	MAPRE1	gi|6912494	30151/5.02	↓	↓	↑	20
8	Proteasome subunit alpha type-5	PSMB5	gi|7106387	26579/4.74	↓	↑	↓	43
9	Heat shock protein 27	HSP27	gi|662841	22427/7.83	↓	↓	↑	13
10	14-3-3 protein beta/alpha	YWHAB	gi|197692221	28209/4.76	↑	↓	↑	28
11	Nuclear chloride channel	CLIC	gi|4588526	27249/5.02	↑	↓	↑	16
12	Serine/threonine-protein phosphatase 6 catalytic subunit isoform b	PPP6B	gi|4506029	35806/5.43	↓	↓	↓	28
13	Splicing factor, arginine/serine-rich 2	SCAF1	gi|62898065	25487/5.12	↑	↑	↑	15
14	Spermidine synthase	SRM	gi|531202	34360/5.21	↓	↓	↓	5
15	Serine/threonine-protein phosphatase PP1-beta catalytic subunit isoform 1	PPP1CB	gi|4506005	37961/5.84	↑	↓	↓	25
16	Uroporphyrinogen decarboxylase	UROD	gi|1322019	41119/5.77	↑	↓	↑	20
17	Ubiquinol-cytochrome c reductase core I protein	UQCRC1	gi|515634	53270/5.94	↑	↑	↑	15
18	Glutathione synthetase	GSS	gi|4504169	52523/5.67	↑	↑	↑	24
19	Vimentin	VIM	gi|62414289	53676/5.06	↓	↓	↓	7
20	Actin, cytoplasmic 1	ACTB	gi|4501885	42052/5.29	↑	↑	↑	29
21	Serum albumin	PRO2044	gi|6650826	30084/7.70	↓	↑	↑	5
22	Thymosin beta-10	TMSB10	gi|339697	5701/6.45	↓	↓	↓	16
23	Transcription factor BTF3 homolog 4	BTF3L4	gi|29789195	17260/5.95	↓	↓	↑	5
24	Superoxide dismutase	SOD1	gi|4507149	16154/5.70	↓	↓	↑	59
25	Stathmin isoform a	STMN1	gi|5031851	17292/5.76	↑	↑	↓	15
26	Inosine triphosphate pyrophosphatase	ITPA	gi|13398328	21827/5.78	↓	↓	↑	18
27	Thioredoxin 1	TXN1	gi|9280551	11971/4.69	↑	↓	↑	49
28	Cofilin-1	CFL1	gi|5031635	18719/8.22	↓	↑	↑	51
